# Functional Time Domain Diffuse Correlation Spectroscopy

**DOI:** 10.3389/fnins.2022.932119

**Published:** 2022-08-01

**Authors:** Nisan Ozana, Niyom Lue, Marco Renna, Mitchell B. Robinson, Alyssa Martin, Alexander I. Zavriyev, Bryce Carr, Dibbyan Mazumder, Megan H. Blackwell, Maria A. Franceschini, Stefan A. Carp

**Affiliations:** ^1^Department of Radiology, Athinoula A. Martinos Center for Biomedical Imaging, Massachusetts General Hospital, Harvard Medical School, Boston, MA, United States; ^2^Massachusetts Institute of Technology Lincoln Laboratory, Lexington, MA, United States; ^3^Massachusetts Institute of Technology, Health Sciences and Technology Program, Cambridge, MA, United States

**Keywords:** diffuse correlation spectroscopy (DCS), cerebral blood flow, fNIRS (functional near infrared spectroscopy), neuroimaging (anatomic and functional), optical neuroimaging

## Abstract

Time-domain diffuse correlation spectroscopy (TD-DCS) offers a novel approach to high-spatial resolution functional brain imaging based on the direct quantification of cerebral blood flow (CBF) changes in response to neural activity. However, the signal-to-noise ratio (SNR) offered by previous TD-DCS instruments remains a challenge to achieving the high temporal resolution needed to resolve perfusion changes during functional measurements. Here we present a next-generation optimized functional TD-DCS system that combines a custom 1,064 nm pulse-shaped, quasi transform-limited, amplified laser source with a high-resolution time-tagging system and superconducting nanowire single-photon detectors (SNSPDs). System characterization and optimization was conducted on homogenous and two-layer intralipid phantoms before performing functional CBF measurements in six human subjects. By acquiring CBF signals at over 5 Hz for a late gate start time of the temporal point spread function (TPSF) at 15 mm source-detector separation, we demonstrate for the first time the measurement of blood flow responses to breath-holding and functional tasks using TD-DCS.

## Introduction

The use of optical methods for neuroimaging, such as functional near infrared spectroscopy (fNIRS), has significantly increased in the last decade (Yücel et al., 2017; Chen et al., 2020). fNIRS, as typically implemented using continuous-wave (CW) approaches (Scholkmann et al., 2014) suffers from two major limitations: the first one is the limited brain sensitivity of CW-fNIRS (Tachtsidis and Scholkmann, 2016), as the majority of the photons have only sampled the scalp and the skull; second, fNIRS methods are based on the measurement of hemoglobin concentration changes, which is only a byproduct of blood flow changes that are driven by neurovascular coupling (Mesquita et al., 2009). Diffuse correlation spectroscopy (DCS) has emerged as a non-invasive optical method for the measurement of tissue blood flow (BF). Using a coherent, near-infrared light source, DCS is able to report a measure of blood flow in tissue, the blood flow index (BF_*i*_), through the analysis of the temporal fluctuations of the measured speckle pattern that forms on the surface of the tissue (Boas and Yodh, 1997). These fluctuations are caused by dynamic scattering events along the photon path, mainly reflecting the movement of red blood cells. The fluctuations of the speckle intensity are quantified by the temporal, intensity autocorrelation function, g_2_(τ), and BF_*i*_ (Boas et al., 1995; Boas and Yodh, 1997) can be estimated by fitting a correlation transport model to the measured g_2_(τ) (Boas et al., 2016). As reported (Selb et al., 2014), DCS has an intrinsic sensitivity to brain perfusion changes that is ∼3x times higher compared to CW-NIRS since the brain blood flow is ∼6x greater than scalp flow while brain hemoglobin concentration is only ∼2x greater than scalp hemoglobin (Steinbrink et al., 2001). However, the light throughput limitations of DCS driven by the need to sample individual speckle fluctuations have been a barrier to realizing this promise. Since the sensitivity region of diffuse optical techniques is dependent upon the paths of the detected photons through the tissue, for neuroscience applications, large source-detector (SD) separations (> 2.5 cm) are needed to improve the sensitivity of the measurement to cerebral blood flow (CBF) and reduce the influence of extracerebral contamination (Buckley et al., 2014). While these larger SD separations are desirable to improve sensitivity, both the signal-to-noise ratio (SNR) and spatial resolution are reduced, which can hinder functional measurements.

To improve upon both of these factors, our group has pioneered the development of DCS in the time domain (TD-DCS) (Sutin et al., 2016). As in TD-NIRS, in TD-DCS photons are selected by their time-of-flight (ToF). This method allows for the rejection of short pathlength photons that travel mostly in extracerebral layers (i.e., scalp and skull), while keeping the longer pathlength photons that travel deeper and reach the brain (Cheng et al., 2018; Mazumder et al., 2021). A major benefit of this ToF selectivity is that shorter SD separations can be used, improving upon the SNR and spatial resolution of the measurements.

However, TD-DCS has stringent requirements on both the laser source and photodetectors. The laser pulse needs to be long enough to provide enough coherence for in-gate photons to interfere, and at the same time, the overall instrument response function (IRF) needs to be short enough to allow effective selection of photons based on their time of flight. This further requires the use of photodetectors with low jitter and a sharp temporal response over multiple decades (i.e., lacking a “diffusion tail”). The importance and impact of these instrumentation related factors have been explored by our group and others through both simulations (Qiu et al., 2018, 2021; Colombo et al., 2019; Mazumder et al., 2021) and experiments (Tamborini et al., 2019; Samaei et al., 2021a).

Previously reported TD-DCS systems (Pagliazzi et al., 2017; Tamborini et al., 2019; Colombo et al., 2020; Samaei et al., 2021b) face limitations with respect to detector efficiency and/or the characteristics of the IRF. To address these limitations, and building on our previous work demonstrating the benefits of DCS measurements at 1,064 nm (Carp et al., 2020; Ozana et al., 2021), as well as simulation studies by our group (Mazumder et al., 2021) and others (Qiu et al., 2018, 2021; Colombo et al., 2019) indicating the importance of optimizing laser source characteristics for TD-DCS, we developed a high-performance, next-generation TD-DCS system employing a custom 1,064 nm laser source and superconducting nanowire single photon detectors (SNSPDs, overall size of 69 × 49.5 × 113 cm for depth, width, and height, respectively) to maximize measurement performance and enable functional brain imaging. The laser source can deliver an optimized, quasi-transform limited ∼300 ps full width half max (FWHM) laser pulse with average power in excess of 500 mW at 1,064 nm. This allows us to take advantage of the full benefits of 1,064 nm operation, which offers several regulatory and intrinsic benefits compared to the 750–850 nm range previously used by DCS systems (Tamborini et al., 2019). At this wavelength, available photon counts (and thus SNR) increase due to higher maximal permissible exposure (MPE) as given by the regulatory standards, lower per photon energy, and lower tissue attenuation, as we have demonstrated previously for continuous-wave DCS (CW-DCS) (Carp et al., 2020; Ozana et al., 2021). Furthermore, the autocorrelation function decay is slower, due to the longer wavelength and lower reduced scattering coefficient, allowing for higher SNR at longer ToFs. Of note, the realization of these benefits has been hampered by the lack of availability of single photon detector suitable for TD-DCS at this wavelength. Silicon single photon avalanche diodes (SPADs) have poor sensitivity beyond 1,000 nm, while indium gallium arsenide (InGaAs) SPADs, which provide good photon efficiency above 900 nm, have undesirable dark count rate (DCR) and afterpulsing characteristics (Ozana et al., 2021). When afterpulsing occurs, more than one electrical pulse per single incident photon is generated. One solution to reduce afterpulsing in InGaAs detectors is to allow the diffusion of the trapped charges by applying long hold off times (∼10 μs). However, this solution is not acceptable for DCS, since the light intensity fluctuations on the order of several microseconds need to be captured for accurate DCS measurements (Jiang et al., 2007). To this end, we have recently shown afterpulsing free superconducting nanowire single photon detectors (SNSPDs) enable effective CW-DCS at 1,064 nm (Ozana et al., 2021). These afterpulsing free detectors demonstrate high photon detection efficiency (> 80%), low DCR [10–100 counts per second (CPS)], high timing resolution (< 100 ps) and rapid reset time (< 50 ns). All these factors led to the choice of SNSPDs as detectors for our next-generation TD-DCS system. A similar approach, though without the ability to customize the laser pulse characteristics has recently been reported by Poon et al. (2022).

In this work, we describe the new TD-DCS system operating at 1,064 nm based on high-precision temporal pulse shaping, SNSPD sensing, and a low-jitter time tagging system. The pulse shaping allows us to optimally address the competing requirements of sufficient coherence and accurate photon selection by ToF that we have detailed in our previous simulation study. System performance metrics (e.g., coherence parameter (β), measurement SNR, etc.) were characterized with phantom measurements. Finally, the sensitivity and SNR of the system in *in vivo* measurements was evaluated in experiments with six human subjects performing tasks to vary both cerebral and systemic perfusion, including superficial blood flow modulation using a tourniquet, breath holding, and functional activation measurements. In particular, this is the first time that TD-DCS has been used to monitor brain perfusion changes in response to functional activation in human subjects.

## Materials and Methods

### Quasi-Transform Limited Pulse Shaping

Precise control over the laser pulse shape is needed to balance photon ToF selectivity, requiring shorter pulses, with the need for sufficient coherence length (several cm, to cover longer paths taken by photons arriving at the detector), which demands larger pulses (Sutin et al., 2016). In addition to these requirements, another design goal we had for the TD-DCS system was to be able to exploit the maximum power per illumination location permitted by the ANSI standards (ANSI Z136.1-2000) at 1,064 nm, ∼100 mW (∼4 times more energy with respect to our previous TD-DCS system operating at 766 nm).

**FIGURE 1:**
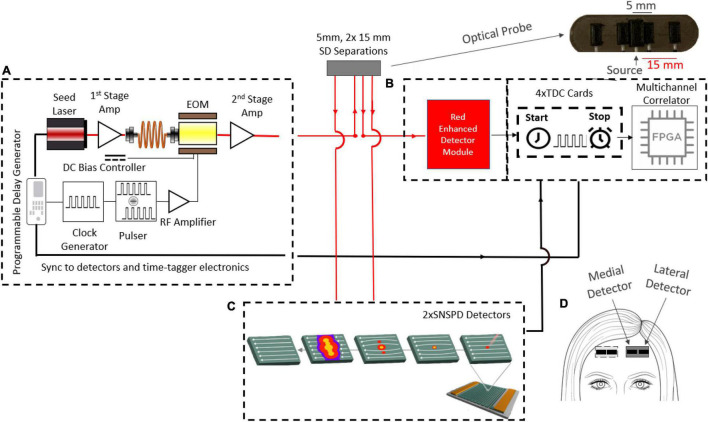
Schematic diagram of the experimental setup. **(A)** Custom 1,064 nm two-stage fiber amplified pulsed laser. The pulsed system can emit 100 mW average power with at a repetition rate of 1–100 MHz and an adjustable pulse FWHM from 150 to 600 ps. The first stage amplifier is fed by the pulsed seed laser and its output is spliced to the EOM input, while the EOM output feeds the second stage amplifier. The second stage output is spliced to a 62.5μm multimode 90:10 fiber splitter. The 90% fiber is finally spliced to the delivery probe and the 10% fiber is used for measuring the instrument response function (IRF). The optical probe consists of a source fiber, two detector fibers at 5 mm (only one was used) and another two detector fibers at 15 mm separation. **(B)** The TD detection module consists of an MPD-RE detector for short separation (5 mm) measurements and **(C)** 2 SNSPD detectors for the long separation (15 mm) measurements. A custom time-tagger with four TDC cards and an FPGA board were used to record the micro times (ToF) and the macro times (absolute times of arrival). **(D)** The DCS optical probe, consists of medial and lateral detectors was attached to either side of the forehead. Measurements during functional tasks were repeated on both sides of the forehead (left and right hemispheres) on each subject.

To these ends, we developed a custom laser system, shown below in Figure 1. The system consists of a 5 mW, 1,064 nm pulsed seed laser (CPDL-S-F-NS-1064, wide pulse (∼600 ps) version, PicoQuant GMBH), two stages of Ytterbium-Doped Fiber Amplifiers (YDFA) (Thorlabs YDFA100P, Cybel MAKO–AMP1064), a programmable delay generator (Micro Photon Devices PSD-MOD), a custom-built RF synchronized picosecond pulse generator, and a Mach-Zehnder 10 GHz bandwidth electro-optic intensity modulator (EOM, NIR-MX-LN-10 from iXblue Photonics). To ensure that both the desired pulse width and the average power are achieved, the Thorlabs YDFA100P is used to amplify the seed pulse going into the EOM and ensure sufficient laser power at the input of the second stage amplifier after the shaping and attenuation by the EOM (i.e., 1 mW minimum input power for the Cybel-MAKO-AMP 1,064). The YDFA 100P is chosen as the pre-amp, since it is able to amplify small signals, while the Cybel-MAKO-AMP 1,064 was chosen to be the second stage amplifier, since it is able to amplify the light up to 1W average power, which enables future multiple illuminations designs. All stages up to the MAKO amplifier use polarization maintaining single mode fibers. The EOM amplitude modulation is controlled by the custom pulse generator. This pulse generator is capable of generating electrical pulses with widths ranging between 150 and 600 ps. By delaying the electrical signal sent to the EOM relative to the laser trigger, different parts of the seed laser pulse are transmitted, with full control of location and width with respect to the original laser pulse (FWHM of 600 ps). In this experiment, 200, 300, and 600 ps FWHM pulse widths were explored. The average laser power output is maintained at the desired 100 mW level by adjusting the second fiber amplifier across the range of tested pulse locations, widths, and repetition frequencies (a repetition frequency of 25 MHz was used for all the results shown in this paper). The use of 100 mW power at 1,064 nm to illuminate the skin is in compliance with the ANSI standards (1 W/cm^2^ over a 3.5 mm aperture as long as the spot size > 1 mm) (Winburn, 2017). The amplified, shaped optical pulse was delivered into a 90:10 splitter using 62.5 μm graded-index multimode fiber (Thorlabs, GIF625). The 90% branch was spliced to the optical probe, while the 10% branch was used to monitor the laser pulse. To ensure eye safety during the experiments, the beam is passed through a holographic diffuser (Edmund Optics) with an 80 degree divergence integrated into the optical probe.

In the probe, light was collected at 5 and 15 mm *SD* separations from the source using single mode fibers (Figure 1), and at the larger separation, to achieve detection of two areas, we used two detection fibers symmetrically positioned around the source. Light from these fibers was detected by 2 SNSPD detectors (Quantum Opus, Opus One) operating at 3.1°K with a direct current of 7 μA, optimized for 1,064 nm operation, yielding a photon detection efficiency of over 80%. While in time-domain very short source-detector separations can be used, we chose a 15 mm SD separation to avoid saturation of the SNSPDs (achieving count rates of ∼1 Mcps, within the linear range of the detectors). The ToF of individual photons (termed micro-time, 10 ps resolution) and the absolute arrival times (termed macro-time, 6.6 ns resolution) of the photons collected by the SNSPD detectors were recorded by a custom FPGA-powered multichannel time-tagger system for time-correlated single-photon counting (TCSPC) (Becker et al., 2005). The high precision time-tagger system consisted of 4 time-to-digital converter (TDC) cards that able to measure time intervals with 10 ps resolution and up to 6.5 M/s conversion rate (Markovic et al., 2013; Tamborini et al., 2015). These were plugged into a custom-developed motherboard that further tagged each photon detection using a 150 MHz on-board clock as described in our previous publication (Tamborini et al., 2019). This allows us to select for late photons that have traveled deeper into the tissue based on their TOFs, then compute auto-correlation curves specifically from these photons. The SM fiber at 5 mm *SD* separation was sent to an MPD RE (Gulinatti et al., 2021) silicon SPAD for pseudo-CW (not time resolved) monitoring of scalp blood flow. At this short SD distance, there were sufficient photons to overcome the very low photon efficiency of the Si-SPAD at 1,064 nm (∼3%).

In this paper we present TD-DCS results at different gates: an early gate starting at the peak of the TPSF, a late gate starting 400 ps after the TPSF peak, and a very late gate starting 600 ps after the TPSF peak. For the phantom measurements we examined three gate widths: 200 ps, 500 ps, and 1 ns. The human measurements used only the 1 ns gate width to maximize the SNR.

First, we measured the temporal stability of the custom-made laser system by measuring the IRF and the temporal point spread function (TPSF) of a homogenous liquid phantom for an hour. Tissue-like optical properties (μ_a_ = 0.14 cm^–1^, μ_s_’ = 7 cm^–1^) were achieved by mixing intralipid and water. The reduced scattering coefficient was estimated by extrapolating the results from a reference frequency domain near-infrared spectroscopy (FD-NIRS) instrument with 8 wavelengths between 670 and 830 nm (MetaOx, ISS) (Carp et al., 2017). The absorption coefficient was estimated to equal that of water at 1,064 nm (Carp et al., 2020). The Brownian diffusion coefficient (D_b_) was measured (at ∼18°C) to be 9⋅10^–9^ cm^2^/s using a CW-DCS laser source at 1,064 nm (CL1064-300-SO, CrystaLaser) and the same SNSPD detectors as for the TD-DCS system. The temperature of the liquid phantom, EOM, seed laser, and RF pulse generator were continuously monitored throughout the measurement.

### Pulse Shaping Optimization

**FIGURE 2:**
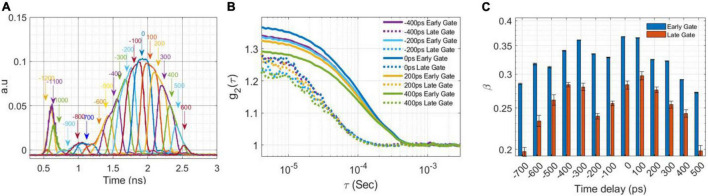
Laser shaping. **(A)** The laser pulses (FWHM of 300 ps at 25 MHz) derived from delaying the RF signal to the EOM with respect to the seed laser by 100 ps increments **(B)** The autocorrelation curve, g_2_ (τ) from an intralipid phantom for a subset of the pulse shaping delays in **(A)** at 1.5 cm *SD* separation calculated for the early and late gates at 200 ps gate width. **(C)** The measured coherence factor, β, as a function of the EOM temporal alignment calculated at early and late gates.

Shaped optical pulses originating from different sections of the seed laser pulse may have different coherence properties. To determine the optimal segment of the seed laser from which to generate the shaped pulse, the EOM opening time was swept from −1,200 to 600 ps relative to the peak of the unshaped pulse with 100 ps steps and 300 ps pulse width, shown in Figure 2A. For each generated pulse, we computed the autocorrelation curve [g_2_(τ)] for a gate width of 200 ps centered on the TPSF peak. The values of the coherence parameter (β) of the g_2_ curves at different delays were used to quantify the coherence of each section of the seed pulse. The achievement of a quasi-transform limited pulse was verified by comparing the coherence length (estimated with the Mach Zehnder interferometer) with the ideal value and also that of the original pulse.

To investigate the effect of pulse width and gate duration on g_2_ coherence contrast (which has a significant impact on measurement SNR), data was taken on a homogenous phantom using pulses of different durations [FWHM of 200, 300, and 600 ps (unshaped)]. The collected data was then post-processed using gate widths of 200 ps, 500 ps, and 1 ns. Further, to demonstrate the better depth sensitivity of a shaped laser pulse (300 ps) vs. unshaped (600 ps) as predicted by simulations, a two-layer phantom solution was made from intralipid (bottom, “fast” layer) and intralipid+methylcellulose (for increased viscosity) (top, “slow” layer) to simulate brain and scalp blood flow, respectively. The optical properties of the top and bottom layers at 1,064 nm were the same, μ_*a*_ = 0.14 cm^–1^, μ_*s*_’ = 7 cm^–1^, while the top layer (slower) Brownian diffusion coefficient was D_*b*_ = 1.3⋅10^–9^ cm^2^/s and the bottom layer’s was D_*b*_ = 9.4⋅10^–9^ cm^2^/s (7.2 times higher). The normalized field autocorrelation, g_1,_ was calculated from the measured intensity auto-correlations, g_2_, for different thicknesses of the upper layer: 0, 5, 10, 15, 20, and 25 mm for both shaped and unshaped pulses to demonstrate the depth sensitivity of the raw measurement.

### Human Tests and Data Analysis

Six healthy volunteers (2 females, 4 males, mean age 24 ± 2 years, all right-handed and non-smokers) were recruited for the study. The study was reviewed and approved by the Mass General Brigham Human Research Committee (IRB #2019P003074). The protocol consisted of a tourniquet pressure modulation, breathing tasks, and functional tasks. For the tourniquet pressure modulation and breathing tasks, the DCS optical probe was attached to one side of the forehead, and to account for potential regional differences between the left and right hemispheres, the side of the forehead on which the probe was placed was alternated across subjects. Measurements during functional tasks were repeated on both sides of the forehead on each subject.

In order to assess the scalp sensitivity of the 1,064 nm TD-DCS system at different time gates, we carried out a pressure modulation task using a medical-grade tourniquet (Baker et al., 2014). The tourniquet was positioned between the eyebrow and the optical probes passing over the superficial temporal, the supratrochlear, and the supraorbital arteries. After a 60 s baseline, the tourniquet was tightened for 60 s to reduce scalp blood flow, and released while acquiring for another 60 s during BFi recovery. The task was repeated twice in each subject. In the breathing tasks, subjects were asked to engage in a series of breath-holding periods alternated by self-paced breathing. The task consisted of 30 s of baseline, followed by 20 s breath-holding repeated 3 times and followed by a final 30 s of recovery. Subjects were instructed to exhale during the 5 s leading up to the task, since breath-holding after exhaling leads to a more rapid increase in CBF, allowing for shorter breath-holding observation periods to see the same relative effect size (Ozana et al., 2021). To functionally activate the pre-frontal cortex subjects were asked to mentally perform subtractions of one-digit numbers from a three-digit number during TD-DCS measurements. The task consisted of a series of a 30 s baseline, followed by the mental arithmetic for 20 s, repeated six times and followed by a final 30 s of recovery period. To show the asymmetric functional activation, we repeated the measurements on both sides of the forehead (positioning the probe 2 cm above the eyebrow).

The human subject data was processed using the TD-DCS analytical model previously published by our group (Tamborini et al., 2019) at 5 Hz. We considered an early gate and a late gate, with a gate width of 1 ns. We chose the wide gate because our simulation work indicates wide gates achieve the best brain measurement SNR, and because results are representative of the gating profile of potential future, lower-cost, detector systems that use time gating instead of high-resolution time-tagging for photon selection. For each subject and each trial, we computed the relative blood flow (rBF_*i*_) time-course, by normalizing BF_*i*_ to the mean value of the pre-trial period.

To evaluate the SNR of the TD-DCS measurements, analysis of the baseline segments of the pressure modulation task was performed. To reduce the influence of cardiac pulsatility on the estimate of the noise in the g_2_(τ) curve, time intervals for analysis were selected at the diastolic interval in the cardiac cycle, identified by the BF_*i*_ fits from the early gate as also done in our previous SNSPD enabled CW-DCS analysis (Ozana et al., 2021). To compare the SNR at different gate start times, the mean g_2_(τ = 4 μs) and the standard deviation of g_2_(τ = 4 μs) were calculated by averaging 50 diastolic g_2_(τ) curves acquired at 5 Hz. Finally, the SNR was calculated by the equation: SNR(*g*_2_(τ)) = *mean*(*g*_2_(τ = 4μ*s*)−1)/*STD*(*g*_2_(τ = 4μ*s*)). The pulsatile CNR was estimated as the contrast between the FFT amplitude at the pulsation frequency and the noise floor, and a clear signal was defined using a threshold of CNR > 4 (Ozana et al., 2021).

## Results

### Quasi-Transform Limited Pulse Shaping

The first goal was to reduce the FWHM of the laser pulse while maintain the original coherence length as much as possible, to achieve a quasi-transformed limited pulse, by selecting the part of the seed laser with the highest coherence. This was obtained by centering the EOM around the original laser peak. Figure 2A shows the shaped optical pulses (FWHM of ∼300 ps) that were sampled with a digital sampling oscilloscope equipped with a 9.5 GHz bandwidth optical input (PicoScope 9321-20) by sliding the timing of the EOM opening window along the original seed laser pulse (Rogers and Gould, 2016). Figure 2B reports the auto-correlation curves corresponding to 5 shaped pulse timings measured on an intralipid phantom using a 200 ps gate width at the early and late gates. Figure 2C shows the coherence factor, β (the height of the intensity auto-correlation curve above 1) for the early and late gates (gate width 200 ps) vs. the temporal alignment of the shaped pulse, confirming the optimal choice of EOM alignment.

**FIGURE 3:**
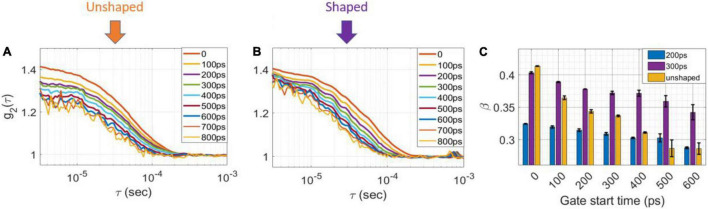
Measurement results with shaped and unshaped pulses for homogenous phantoms. The intensity autocorrelation curves (g_2_) of **(A)** unshaped and **(B)** 300 ps FWHM for the homogenous phantom at different gate start times, 200 ps gate width. **(C)** The coherence factor, β for 200 ps FWHM (blue), 300 FWHM (magenta) and unshaped (orange) pulses as a function of gate start time of homogenous phantom at 200 ps gate width.

Another advantage of shaping the pulse is achieving a nearly transform limited pulse enabling a higher degree of coherence between the detected photons at later gates. Figures 3A,B show averaged g2 curves derived from 100 M photon detections at a separation of 1.5 cm for (a) unshaped and (b) shaped (300 ps FWHM) laser pulses at various gate start times and 200 ps gate width. As shown in Figure 3A, for the unshaped pulse, β decreases from 0.41 for early gates to 0.28 for very late gates, while in the shaped pulse, Figure 3B β decreases only from 0.42 to 0.34 over the same range of gate timing. As shown in Figure 3C, for a 200 ps FWHM pulse the coherence factor is significantly lower compared to the 300 and 600 ps FWHM pulses at the same time gates (with 200 ps gate width), indicating the coherence length of the 200 ps pulse is lower (as expected due to the narrower pulse). A summary of the β values as a function of gate start time and gate width are shown in Table 1. These experimental results were in good agreement with the direct interferometric measurement of the coherence length (lc). At ∼300 ps FWHM pulse width, the pulse was nearly transform-limited, exhibiting a lc = 5 cm, which is close to the unshaped seed laser’s coherence length of 6 cm.

**TABLE 1:** β as a function of gate start time and gate width for shaped (300ps FWHM) and unshaped pulses (∼600 ps FWHM).

Shaped/unshaped	Gate width (ps)	β early gate	β late gate	β very late gate
Shaped	200	0.42	0.37	0.34
Unshaped	200	0.41	0.31	0.28
Shaped	500	0.29	0.26	0.25
Unshaped	500	0.25	0.22	0.21
Shaped	1,000	0.24	0.23	0.22
Unshaped	1,000	0.20	0.20	0.18

### Improved Depth Sensitivity *via* Pulse Shaping

**FIGURE 4:**
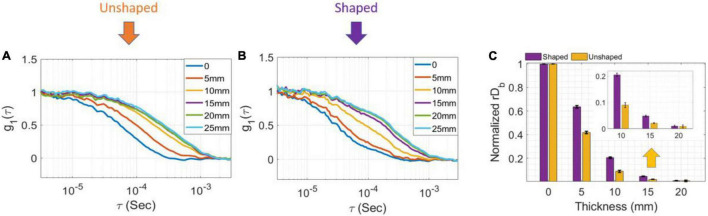
Measurement results with shaped and unshaped pulses for two-layer phantoms. **(A)** The electric field autocorrelation function g_1_, for different thicknesses of the top layer in the two-layer phantom while the laser was unshaped and **(B)** shaped at very late gate start time and 1 ns gate width. **(C)** The min-max normalized rD_*b*_ as a function of the different thicknesses (error bars are stdev).

To evaluate the dependence of sensitivity to depth on the pulse shape, we calculated the electric field autocorrelation functions, g_1_ (to normalize for the changes in β) from measurements taken at several top layer thicknesses in two-layer phantoms. Figure 4A (unshaped pulse) and Figure 4B (300 ps FWHM shaped pulse) show a comparison of the g_1_ curves for several layer thicknesses for the very late gate with a 1 ns gate width. While in the unshaped case, the curves overlap for superficial thicknesses of 15, 20, and 25 mm, for the shaped pulsed the contrast is higher at every depth, and only a superficial layer of 20 mm is indistinguishable from a 25 mm layer, as expected due to the more effective deep photon selection achievable with the shorter pulse (Mazumder et al., 2021). For ease of comparison, we min-max normalized the recovered rD_*b*_ values between those at 0 and at 25 mm superficial layer thickness, respectively (assumed to represent the pure bottom and top layers, respectively)—these normalized values are shown in Figure 4C on a scale from 0 to 1. The rD_*b*_ values at 25 mm are thus, by definition, equal to 0 so they are not shown to improve the clarity of the figure.

Finally, we measured the stability of the TD DCS laser system over 1 h (laser pulse FWHM of 300 ps). The FWHM standard deviation of the IRF and TPSF curves was 2% (100 ± 2%). The standard deviation of the 99% width of the pulse (1/100 value of the TPSF peak) was 2% as well for both TPSF and IRF. Temperatures at different locations during the experiment were seen to exhibit low variability during the measurement, with the temperature of the intralipid solution, EOM, seed laser, and RF generator equal to 17.97 ± 0.09°C, 20.99 ± 0.07°C, 21.36 ± 0.13°C, and 19.10 ± 0.13°C, respectively.

### Human Test Results

**FIGURE 5:**
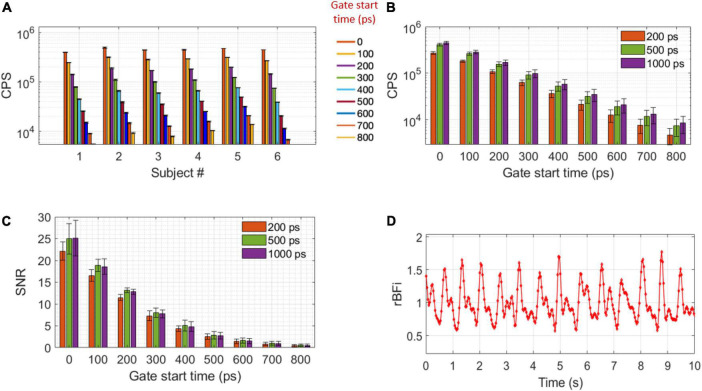
CPS and SNR. **(A)** CPS of all the subjects at gate start times between 0 and 800 ps for a 1,000 ps gate width. Error bars represents temporal STD of the pressure modulation task baseline CPS (between 10 and 50 s). **(B)** CPS and **(C)** SNR at 200, 500, and 1,000 ps gate widths as a function of gate start time, error bars represent STD of all the subjects. **(D)** An example of BF_*i*_ acquired at 30 Hz at late gate in subject # 3 (g_2_ SNR = 5.8). A Butterworth bandpass filter of 0.1–5 Hz was applied to the data.

For traditional NIRS wavelengths, melanin represents a significant component of the optical absorption of tissue, and for darker skin individuals, limits the usability and accuracy of optical technologies. At 1,064 nm, melanin absorption is 73% lower than at 755 nm, while the total absorption change due to hemoglobin and water is negligible (Tanghetti and Jennings, 2018; Carp et al., 2020). On one subject with dark skin (subject #5) we confirmed similar light attenuation as on the lighter skin subjects, as shown in Figure 5 at different start gate times. The average total CPS (integrated over the whole TPSF) per SNSPD channel was 1.13 ± 0.47 Mcps. With a gate width of 1,000 ps and a source detector (*SD*) separation of 1.5 cm, average count rates for different gate start times were: 447 ± 32 kcps at the early gate, 58 ± 14 kcps at a late gate and 21 ± 7 at a very late gate. These numbers are reported in Figure 5B as a function of gate start time, together with CPS at gate widths of 200, 500, and 1,000 ps.

To assess the SNR of the TD-DCS, the BF_*i*_ trace calculated at 5 Hz and peak of the TPSF gate start time is used to identify the diastolic point in the cardiac cycle. During rest, fifty g_2_(τ) curves corresponding to the diastolic point of the arterial pulsation are averaged together for each gate start time and three gate widths (200 500, and 1,000 ps) to evaluate the SNR of the g_2_ curve. As shown in Figure 5C, the SNR is higher when considering a 500 ps gate width with respect to a 200 ps gate width: 25.0 ± 3.5 vs. 22.1 ± 2.1 at early gate and 5.1 ± 1.1 vs. 4.3 ± 1.1 at late gates, respectively. However, there was no statistically significant difference in SNR between 500 ps and 1,000 ps gate widths (25.0 ± 4.0 and 4.8 ± 1.0 at early and late gates, respectively). It is worth noting that our system was able to obtain relatively high g_2_(τ = 4 μs) SNR at the late gate (4.8 ± 1.1) to resolve the functional response. As a result, we were able to extract the pulsation (since in these cases the pulsatile CNR was > 4) at a high sample rate in all subjects (5 Hz). An example of the pulsation at high sample rate is shown in Figure 5D.

**FIGURE 6:**
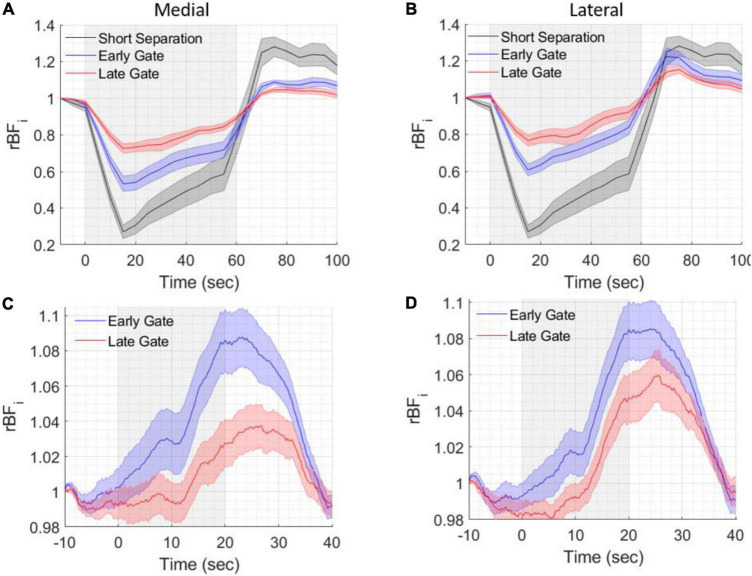
Grand averages of six healthy volunteer test results during pressure modulation and breathing trials. BF_*i*_ changes during the different tasks were averaged across all included trials and all subjects. The gray shaded areas indicate the periods of pressure modulation **(A,B)** or breath holding **(C,D)**. **(A)** BF_*i*_ changes during pressure modulation (60 s) at different short *SD* separation (5 mm, pseudo-CW) and 15 mm *SD* separation and early and late gates at medial and **(B)** lateral spatial locations. **(C)** BF_*i*_ changes during breath holding (20 s) at 15 mm *SD* separation and early and late gates at medial and **(D)** lateral spatial locations. Shading around the lines represents the standard error of the six subjects.

Normalized BFi timecourses during the tourniquet pressure modulation are shown in Figures 6A,B, averaged across all measurements for medial and lateral locations, on all subjects. The highest reduction in BFi due to pressure was found at the 5 mm *SD* separation (57 ± 6%) and consistently, a lower reduction was found at 1.5 cm at both early and late gates, with reductions of 37 ± 6% and 29 ± 6% at early gates and of 20 ± 4% and 18% ± 4% at late gates, indicating a lower scalp contamination for late gates (medial and lateral, respectively). The lower reductions at late gates were statistically significant with respect to both the short separation (5 mm) and the early gates at 15 mm (two-tailed paired sample *t*-test, *p* < 0.05).

As shown in Figures 6C,D (averages of medial and lateral locations across all trials and all subjects), during the 20 s of breath holding blood flow increased due to the transient hypercapnic hypoxia, then slowly return to baseline during the breathing period. We observed higher and earlier blood flow changes at early gates both on the lateral and medial locations. These results are in agreement with our previous SNSPD enabled CW- DCS studies (Ozana et al., 2021), where the response at the short SD separation was higher than the response at longer SD separations.

**FIGURE 7:**
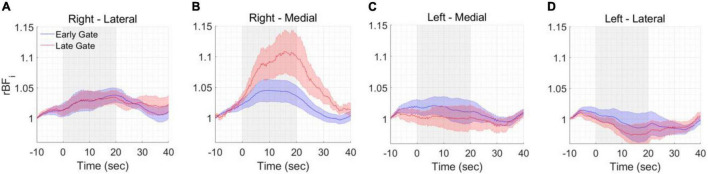
Functional measurements. BF_*i*_ changes during the different tasks were averaged across all included trials and all included subjects at the **(A)** right lateral, **(B)** right medial, **(C)** left medial and **(D)** left lateral locations. A statistically significant response was found at the right-medial location. The shaded area represents the standard error of the six subjects. The gray area represents the duration of the task.

The functional experiments were conducted at two spatial locations, medial and lateral and repeated on the left and right forehead. The rBFi was computed at 5 Hz to fully sample the cardiac pulsation before averaging and avoid it aliasing into the averaged signal as noise. Gate start times ≥ 500 ps from the peak of the TPSF were found to be too noisy at a 5 Hz sampling rate. Figure 7 reports the group averaged responses across all the subjects and all the mental arithmetic task repetitions (a total of 36 trials) for each measured location at early and late gates (a 100 point, i.e., 20 s. moving average was applied). We found significant increases of BFi with the stimulus in the right medial location, when considering late gates, and no or smaller responses in all other locations, Figure 7. Statistically significant differences (*p* < 0.05, two-tailed, paired sample *t*-test) between rBFi at the early and late gates were found after the start of the stimulus at the active location (right medial). No statistically significant differences were found between responses at early and late gates in all the other locations.

## Discussion and Conclusion

We have previously demonstrated the use of SNSPD detectors to effectively implement CW-DCS measurements at 1,064 nm for CBF measurements (Carp et al., 2020; Ozana et al., 2021). Recently, a time gated SNSPD enabled DCS system at 1,064 nm was also demonstrated in a case study on a patient with severe traumatic brain injury and showed good correlation of the DCS measurement with invasive perfusion monitoring (Poon et al., 2022). To our knowledge, in this report we present for the first time an optimized TD-DCS system with sufficient SNR at a late gate to enable brain functional activation measurements. It is also the first report using an EOM to provide a quasi-transform limited laser pulse of optimal length (300 ps FWHM). In our previous Monte Carlo simulation study (Mazumder et al., 2021), we showed that TD-DCS sensitivity is strongly dependent on the IRF shape. Among the different simulated shapes, a ∼300 ps quasi-Gaussian pulse was found to be the optimal. This is in good agreement with the experimental results reported in this paper.

Through the use of the dual stage amplification and custom laser shaping, together with SNSPD detection, we were able to fully realize the promise of long-wavelength (1,064 nm) operation (in comparison, the system reported by Colombo et al. (2020) was limited to shorter wavelengths near 1,000 nm where the attenuation is significantly higher than at 1,064 nm and used an InGaAs photomultiplier tube with 2% quantum efficiency). The increased SNR allows for the measurement of clear arterial pulsation at an acquisition rate of > 5 Hz at the late gate and 1.5 cm separation in all subjects. The late gate provided good discrimination between the cerebral and extracerebral BFi changes for functional measurements. During pressure modulation, the reduction was only 23 ± 4% at the 1.5 cm separation late gate compared to 57 ± 6% at the short (0.5 cm) separation, indicating a substantial degree of CBF sensitivity, thus giving confidence that the signals measured at late gates are derived from cerebral signals. We also note that our results matched the recent simulation study (Wu et al., 2022) that found lower extracerebral thickness at the lateral side of the forehead results in higher sensitivity with respect to more medial locations. This is in good agreement with our pressure modulation task results where we found a 6.7 ± 2.8% greater reduction with pressure modulation in the medial locations compared to the lateral locations indicating somewhat higher extracerebral contribution. However, the difference is small, and confirms the effectiveness of CBF monitoring using 1,064 nm TD-DCS at all relevant forehead locations.

Functional activation results also matched reports in the fNIRS field for mental arithmetic tasks. As reviewed (Soltanlou et al., 2018), subtraction tasks revealed greater activation of the right prefrontal cortex during subtraction compared to rest. This report is in good agreement with our current functional results. Functional measurements using TD-DCS offer two important advantages compared with typical CW-fNIRS implementations. First, the use of time gating removes the requirement for large source-detector separations, thus offering the opportunity to increase spatial resolution and enabling the use of dense optode arrays. Second, no separate short separation channels are needed for systemic physiology regression as the shallow sensitivity measurement needed can simply be performed by computing superficial tissue BF_*i*_ timecourses using the early gate. This further simplifies probe designs and enables increased optode density.

The use of SNSPDs does pose a limitation from the point of view of covering large areas of the head with optode arrays. While SNSPD units with 16 channels or more are beginning to be offered, another alternative may be offered by high channel count gated InGaAs SPAD arrays, currently under development (Blackwell et al., 2020). Compared to seminconductor SPADs, another limitation of SNSPDs is the bulk and the noise of current systems. Future development of more compact SNSPD systems with lower acoustic and thermal footprint may facilitate measurements in clinical space and other environments where the noise and heat dissipation of current systems may be inconvenient.

## Data Availability Statement

The original contributions presented in this study are included in the article/supplementary material, further inquiries can be directed to the corresponding author.

## Ethics Statement

The study was reviewed and approved by the Mass General Brigham Human Research Committee (IRB #2019P003074). The patients/participants provided their written informed consent to participate in this study.

## Author Contributions

NO, MAF, and SAC conceived the project, designed the experiments, and co-wrote the manuscript. NO carried out the *in vivo* and *in vitro* experiments and analyzed the data. NL co-designed the optical system. MR co-designed the hardware integration. AM and AIZ co-carried out the human experiments. BC assisted the development of analysis software. MBR, MR, and DM discussed and consulted on the optical design, data analysis, comparison with simulations, and the manuscript results. All authors contributed to the article and approved the submitted version.

## Conflict of Interest

MAF has a financial interest in 149 Medical, Inc., a company developing DCS technology for assessing and monitoring cerebral blood flow in newborn infants. MAF’s interests were reviewed and managed by Massachusetts General Hospital and Partners HealthCare in accordance with their conflicts of interest policies. The remaining authors declare that the research was conducted in the absence of any commercial or financial relationships that could be construed as a potential conflict of interest.

## Publisher’s Note

All claims expressed in this article are solely those of the authors and do not necessarily represent those of their affiliated organizations, or those of the publisher, the editors and the reviewers. Any product that may be evaluated in this article, or claim that may be made by its manufacturer, is not guaranteed or endorsed by the publisher.
